# INSM1 increases N-myc stability and oncogenesis via a positive-feedback loop in neuroblastoma

**DOI:** 10.18632/oncotarget.5485

**Published:** 2015-10-01

**Authors:** Chiachen Chen, Mary B. Breslin, Michael S. Lan

**Affiliations:** ^1^ The Research Institute for Children, Children's Hospital, New Orleans, LA 70118, USA; ^2^ Laboratory of Diana Helis Henry Medical Research Foundation, New Orleans, LA 70119, USA; ^3^ Department of Pediatrics, Louisiana State University Health Sciences Center, LA 70112, USA; ^4^ Department of Genetics, Louisiana State University Health Sciences Center, LA 70112, USA

**Keywords:** neuroblastoma, N-myc, INSM1, invasion, transformation

## Abstract

*Insulinoma associated-1* (*IA-1/INSM1*) gene is exclusively expressed during early embryonic development, but has been found to be re-expressed at high levels in neuroendocrine tumors including neuroblastoma. Using over-expression and knockdown experiments in neuroblastoma cells, we showed that INSM1 is critical for cell proliferation, BME-coated invasion, and soft agar colony formation. Here, we identified *INSM1* as a novel target gene activated by N-myc in N-myc amplified neuroblastoma cells. The Sonic hedgehog signaling pathway induced INSM1 by increasing N-myc expression. INSM1 activated PI3K/AKT/GSK3β pathways to suppress N-myc phosphorylation (Thr-58) and inhibited degradation of N-myc. Inversely, N-myc protein bound to the E2-box region of the INSM1 promoter and activated INSM1 expression. The invasion assay and the xenograft nude mouse tumor model revealed that the INSM1 factor facilitated growth and oncogenesis of neuroblastoma. The current data supports our hypothesis that a positive-feedback loop of sonic hedgehog signaling induced INSM1 through N-myc and INSM1 enhanced N-myc stability contributing to the transformation of human neuroblastoma.

## INTRODUCTION

Human neuroblastoma (NB) is the most common extracranial childhood tumor arising from the sympathetic nervous system [1, 2]. Amplification of N-myc occurs in roughly 30% of NB patients as well as in a number of other childhood and adult tumors and is strongly correlated with advanced stage disease and poor outcome [3–5]. The Sonic hedgehog (Shh) signaling pathway plays a vital role during early embryonic development. In brain tumors including medulloblastoma (MB) and glioblastoma (GB), Shh plays an important role in stem cell renewal and development [6–8]. NB, an embryonal tumor of the sympathetic nervous system was shown to express high levels of key molecules in the Shh signaling cascade such as SMO and Gli [9]. Blocking of Shh signaling with a SMO inhibitor resulted in a reduction in cell viability with a large portion of the NB cells arresting in G_0_/G_1_ phase of the cell cycle and undergoing apoptosis. Gli2 siRNA treatment decreased cyclin D1 and prevented NB cell growth [7]. Shh signaling stimulates high expression of N-myc in the brain and skin [10] as well as in granular cell progenitors, where N-myc is a main conveyor of Shh mitogenic activity [11].

Insulinoma associated-1 (IA-1/INSM1) was originally cloned from an insulinoma subtraction library [12]. The *INSM-1* gene is expressed strictly during early embryonic development of neuroendocrine (NE) tissues and is re-expressed, at high levels in NE tumors such as NB, MB, RB, pituitary tumor, medullary thyroid carcinoma, and pheochromocytoma [13]. These types of tumors are derived from granule neuron precursors and precursors of the sympatho-adrenal (SA) lineage [7, 14]. Using an *Insm1* gene ablation study, Insm1 was shown to be a crucial component of the transcriptional network that controls differentiation of the SA lineage [15]. Studies revealed that the induction of Insm1 expression in the developing brain correlates with areas where neurogenesis occurs, such as the external granule cell layer of the developing cerebellum, the dentate gyrus of the postnatal hippocampus, the ventricular zone, and, in particular, the subventricular zone of the neocortex [16]. Interestingly, amplification and expression of the *N-myc* gene is the predominant marker for aggressive NB and MB, and correlates with poor prognosis [17]. In this study, we showed that INSM1 possesses extra-nuclear activity to activate the PI3K/AKT signaling pathway that blocks GSK3β activity. Additionally, N-myc acted as an upstream activator for INSM1 and INSM1 expression was crucial to stabilize N-myc protein contributing to NB cell growth and transformation. We further dissected the close relationship of the Shh pathway, INSM1, and N-myc expression in NB cells. Our results revealed a positive-feedback loop that resulted from an increase in N-myc stability through INSM1 activation of the PI3K/AKT signaling pathway thus resulting into NB cell growth, invasion, and transformation. The current data supports our hypothesis that the Shh signal induced INSM1 through N-myc and contributed to the pathobiology of high-risk NBs.

## RESULTS

### Shh increases INSM1 expression and NB cell viability

**Figure 1:**
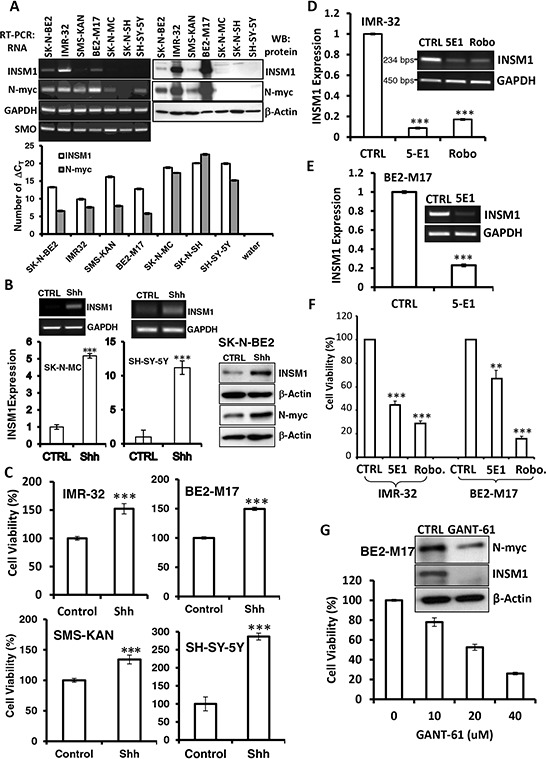
Shh induced INSM1 expression and proliferation in NB cells **A.** Comparative RNA expression of INSM1, SMO, N-myc and GAPDH in seven NB cell lines, SK-N-BE2, SK-N-MC, SH-SY-5Y, BE2-M17, IMR-32, SMS-KAN, and SK-N-SH were performed with standard RT-PCR and/or real time PCR (number of ΔC_T_ was presented) analyses. Western blot analyses of INSM1, N-myc and β-actin were performed using a specific antibody sequentially after striping the same blot. **B.** SK-N-MC, SH-SY-5Y, and SK-N-BE2 cells were stimulated with recombinant Shh-N (1 μg/ml) for three days. Expression levels of INSM1 and N-myc were determined by RT-PCR, quantitative real-time PCR (***p < 0.001), and Western blot analysis. Data are represented as mean ± SEM. **C.** Four cell lines, IMR-32, BE2-M17, SMS-KAN, and SH-SY-5Y were stimulated with recombinant Shh-N (1 μg/ml) for three days and the cell proliferation was measured using a MTS assay. The MTS assay readings (A_490_) were compared with the PBS control (CTRL). Data are represented as mean ± SEM. **D.** IMR-32 cells were treated with a Shh inhibitor (robotnikinin) (0.5 μg/well) or a neutralizing antibody (5E1) (1 μg/well) for three days. RNA transcripts were subjected to RT-PCR and quantitative real-time PCR analyses. **E.** BE2-M17 cells were treated with 5E1 antibody to inhibit Shh signaling. Data are represented as mean ± SEM. **F.** Both IMR-32 and BE2-M17 cells were incubated with Shh neutralizing antibody (5E1) or Shh inhibitor (Robo.) for 3 days. Cell growth was inhibited as shown by the MTS assay (**p < 0.01; ***p < 0.001). Data are represented as mean ± SEM. **G.** BE2-M17 NB cells were incubated with GANT-61 (0–40 μM) for 3 days. The cell viability was monitored using an MTS assay. The expression levels of N-myc and INSM1 in the cells treated with 20 μM of GANT-61 was determined by Western blot analysis. Actin was used as a loading control.

INSM1 expression is restricted to embryonic NE tissues and tumors. The strong association of INSM1 expression with childhood tumors including NB was reported, exemplifying the current embryonic tumor model [17, 18]. The Shh signaling pathway and N-myc expression play critical roles in the proliferation and differentiation of NB cells and NE tumors [19, 20]. All of the NB cells express the *Smoothened* (*SMO*) gene with varying degrees of intensity. *INSM1* gene expression can be detected in SK-N-BE2, BE2-M17, and IMR-32 cells, whereas N-myc protein expression is consistent with INSM1 except in the SMS-KAN cell line (Fig. 1A). A small amount of N-myc transcripts were detected in SK-N-MC and SH-SY-5Y however no protein was detected. When we stimulated the SK-N-MC, SH-SY-5Y, or SK-N-BE2 cells with recombinant Shh-N (1 μg/ml) for three days, we found that Shh induces INSM1 expression at both the RNA and protein levels (Fig. 1B). Additionally, Shh also induces N-myc protein expression in the SK-N-BE2 cells. Consistently, the recombinant Shh-N (1 μg/ml) enhanced NB cell viability in IMR-32, BE2-M17, SMS-KAN, and SH-SY-5Y cells (Fig. 1C). In contrast, when we suppressed Shh signaling activity using the Shh inhibitor, robotnikinin or a neutralizing antibody (5E1), both inhibitors bound to Shh and blocked the signaling in either IMR-32 or BE2-M17 cells. The result showed that blocking Shh signaling caused dramatic inhibition (75–80%) of endogenous INSM1 messenger RNA (Fig. 1D and 1E). The Shh inhibitor not only blocked the *INSM1* gene expression, but also inhibited the NB cell viability in a MTS assay (Fig. 1F). We performed a study to treat NB cells with a Shh inhibitor, GANT-61. BE2-M17 cells were subjected to the Shh inhibitor treatment that blocks Gli-binding and transcriptional activity. GANT-61 inhibited growth of the BE2-M17 cells in a dose-dependent manner and down regulated both N-myc and INSM1 expression (Fig. 1G). At 40 μM concentration, only 20% of the cells survived the drug treatment. Therefore, the Shh signaling pathway positively correlated with N-myc and INSM1 expression. The association of Shh with N-myc and INSM1 expression contributes to NB cell viability.

### N-myc binds and activates the E2-box of INSM1 promoter

**Figure 2:**
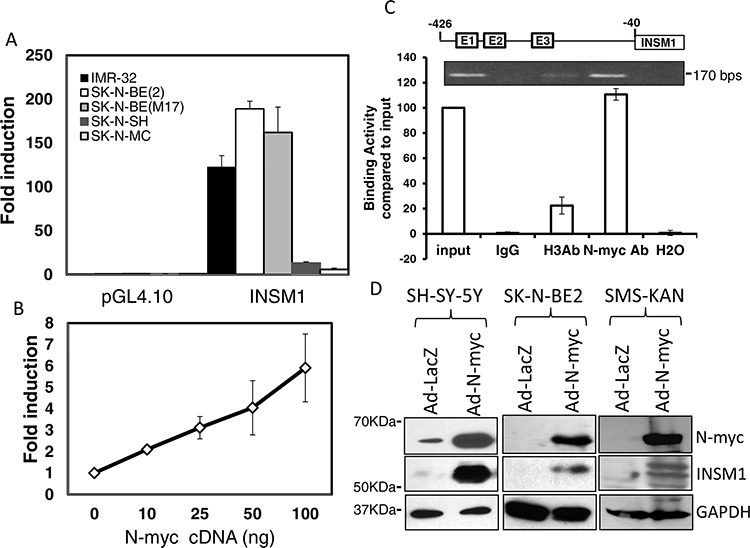
N-myc interacted with the E2-box region in the INSM1 promoter and activated endogenous INSM1 expression in NB cells **A.** Transient transfection of 1.7 kb INSM1 promoter-linked luciferase versus pGL4.10 promoterless vector was performed in five NB cell lines. Data are represented as mean ± SEM. **B.** INSM1 promoter activity was measured with increasing concentrations of N-myc cDNA expression plasmid in SK-N-SH (N-myc negative) cells. All transfections were normalized with TK-Renilla. The graph represented experiments performed in triplicate on 3 separate occasions. Data are represented as mean ± SEM. **C.** A ChIP assay was performed using cross-linked chromatin from BE2-M17 cells. N-myc bound to the E2-box region on the human INSM1 promoter. BE2-M17 chromatin was incubated with rabbit IgG, rabbit anti-Histone-H3 antibody, or rabbit anti-N-myc (B.8.4) antibody. The bound DNA was compared with 2% of input DNA using PCR amplification with primers spanning the human INSM1 promoter E2-box region (170 bps). **D.** SH-SY-5Y, SK-N-BE2, and SMS-KAN cells were infected with Ad-N-myc or Ad-LacZ for 3 days to induce endogenous INSM1 expression. N-myc and INSM1 expression levels were measured by Western blot analysis. GAPDH was used as loading control.

It was reported that N-myc could act downstream of the Shh/SMO signaling pathway during cerebellar granule neuronal precursor cell growth [11, 21]. In the current study, we found that not only Shh induced the *INSM1* gene via N-myc, but N-myc stimulated *INSM1* gene expression directly by binding to the E2-box element on the INSM1 promoter. We transfected the INSM1 promoter-linked luciferease reporter into five NB cell lines, the results were consistent with the N-myc expression pattern (Fig. 2A). The higher luciferase activities were detected in those cells with high N-myc expression. Conversely, extremely low levels of luciferase activity was detected in two cell lines that have no or low N-myc expression, SK-N-SH and SK-N-MC, and corresponded to the quantitative real time PCR result (Fig. 1A). The INSM1 promoter responded in a dose-dependent manner to stimulation by N-myc (Fig. 2B). In Fig. 2C, several pairs of primers spanning the INSM1 promoter region (including E1, E2, E3-box) were designed. The primer pairs spanning the E2-box showed a positive ChIP assay suggesting that the endogenous N-myc protein binds directly to the E2-box region. When we infected three INSM1-negative or low-expressing NB cell lines, SH-SY-5Y, SK-N-BE2, and SMS-KAN with Ad-N-myc virus, the induced endogenous INSM1 protein was readily detected by Western blot analyses (Fig. 2D). However, there is a discrepancy between the expression of N-myc in Ad-LacZ infected SK-N-BE2 and SMS-KAN cells in contrast to the Fig. 1A. It is probably due to the exposure time and the relative sensitivity.

### INSM1 increases N-myc levels by activating PI3K/AKT/GSK3β signaling pathways and enhances N-myc stability

**Figure 3:**
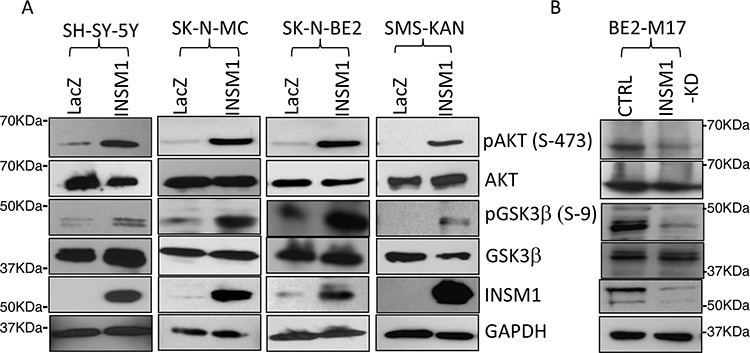
INSM1 activated PI3K/AKT/GSK3β signaling pathways **A.** SH-SY-5Y, SK-N-MC, SK-N-BE2 and SMS-KAN cells were infected with Ad-LacZ or Ad-INSM1 virus for three days. Total cell lysates were subjected to Western blot analyses for the indicated antibody. **B.** BE2-M17 cells were transfected with INSM1 siRNA for 48 hours. The amounts of phosphorylated forms of AKT (pAKT), or GSK3β (pGSK3β) were detected with Western blot analyses. The same blots were stripped and re-probed with antibodies specific for total proteins of AKT, GSK3β, and GAPDH. The data shown represents three independent experiments.

The current data shows that INSM1 expression is stimulated by Shh signaling via the N-myc oncogenic factor in NB tumors. Previously, we reported that INSM1 possessed extra-nuclear activity of enhancing AKT phosphorylation via the insulin receptor signaling pathway [22]. We employed both over-expression and knockdown of INSM1 in NB cells to examine whether the PI3K/AKT/GSK3β pathways could be modulated in NB cells. As shown in Fig. 3A, over-expression of INSM1 in the cells lacking INSM1 expression (SH-SY-5Y, SK-N-MC and SMS-KAN) or with a low level of INSM1 (SK-N-BE2) induced AKT phosphorylation and sequentially activated AKT kinase to inactivate GSK3β kinase by phosphorylation of GSK3β. In the cells with high levels of INSM1 expression, BE2-M17, we performed the knockdown of endogenous INSM1 expression. Consistently, the levels of phospho-AKT and phospho-GSK3β decreased (Fig. 3B). The result supports that INSM1 expression in NB cells activated the PI3K/AKT/GSK3β pathways and contributed to the growth of NB cells.

**Figure 4:**
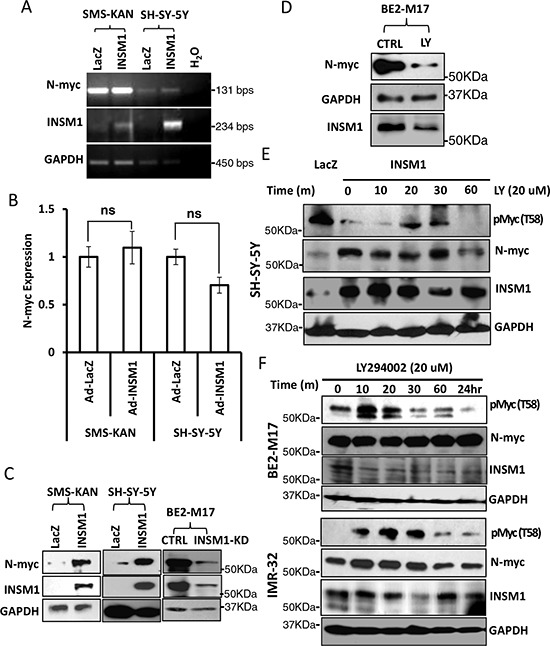
INSM1 increased N-myc protein levels through activation of PI3K/AKT/GSK3β signaling pathways and stabilized N-myc **A.** SMS-KAN and SH-SY-5Y cells were infected with Ad-LacZ or Ad-INSM1 virus for three days. N-myc RNA levels were determined by standard RT-PCR or **B.** Quantitative real-time PCR. “ns” represents not significant. The result indicated that INSM1 had no effect on stimulating *N-myc* gene expression. **C.** SMS-KAN and SH-SY-5Y cells without endogenous INSM1 expression were infected with Ad-INSM1 virus, whereas BE2-M17 cells were transfected with INSM1 siRNA oligomer to knockdown the endogenous INSM1 expression. After 3-days infection or transfection, N-myc protein levels and INSM1 expression were determined by Western blot analyses. The blot was re-probed with anti-GAPDH antibody as an internal control. **D.** BE2-M17 cells were treated with the PI3K/AKT pathway-specific inhibitor (LY294002, 20 nM) for 2 days. N-myc and INSM1 expression levels were measured by Western blot assay. **E.** SH-SY-5Y cells were infected with Ad-LacZ or Ad-INSM1 virus for three days and then the infected cells were incubated with 20 nM LY294002 for the indicated time points. **F.** BE2-M17 and IMR-32 cells were directly treated with 20 nM LY294002 for the indicated time points and then phospho-Myc (T58), N-myc, INSM1, and GAPDH expression were determined by Western blot assay.

Shh signaling leads to stabilized N-myc protein with the activation of PI3K/AKT/GSK3β pathways, which blocks phosphorylation of N-myc at Thr-50. Inactivation of Shh activates GSK3β activity leading to N-myc phosphorylation that enhances final degradation [23]. In parallel, INSM1 is capable of activating PI3K/AKT and inhibits GSK3β kinase, which is responsible for Thr58/Thr50 of C-myc/N-myc phosphorylation that triggers the degradation of Myc protein. As shown in Fig. 4C, in Ad-INSM1 virus infected SMS-KAN and SH-SY-5Y cells, INSM1 increased the amount of N-myc protein but had no effect on N-myc mRNA levels (Fig. 4A and 4B). However, a discordance was noted between the N-myc RNA transcript and protein levels in the absence of INSM1 expression in SMS-KAN cells. Over-expression of INSM1 in SMS-KAN cells enhanced N-myc stability and protein levels (Fig. 4C). Alternatively, knockdown of endogenous INSM1 expression in BE2-M17 cells dramatically decreased N-myc protein levels. These results demonstrated that in the presence of INSM1, N-myc protein levels increased, but not via direct up-regulation of *N-myc* gene expression. It is potentially through the inhibition of the existing N-myc degradation in NB cells. We further assessed N-myc stability using the specific inhibitor of PI3K/AKT signaling pathway, LY294002. The results showed that the PI3K/AKT inhibitor decreased the amount of N-myc that was stabilized by INSM1 (Fig. 4D). We treated endogenous high INSM1 expression cells (BE2-M17 and IMR-32) with LY294002 and found that after 10 minutes treatment Thr-58 phosphorylated Myc protein increased. This result shows that when PI3K/AKT signaling is blocked by LY294002, the GSK3β activity increases and Thr58/Thr50 phosphorylation of Myc protein increases (Fig. 4F). However, the PI3K/AKT inhibitor may not directly abrogate the amount of N-myc. The N-myc stability depends upon entering the ubiquitin degradation pathway. A consistent result was observed in the NB cells without endogenous INSM1. SH-SY-5Y cells were infected with Ad-INSM1 virus for three days and then treated with LY294002 for the indicated time points. Over-expression of INSM1 decreased the amount of phosphorylated Myc protein (Thr-58). However, after 20–30 minutes of LY294002 treatment, the phosphorylated Myc protein level increased suggesting the activation of GSK3β activity (Fig. 4E). In summary, our results correlate that INSM1 enhanced N-myc protein levels through activating the PI3K/AKT pathway and sequentially decreasing the level of phosphorylated Thr-58 Myc protein due to the GSK3β kinase inactivation [24]. Therefore, we conclude that a positive-feedback loop is generated via INSM1 increasing N-myc protein levels through the PI3K/AKT/GSK3β signal pathways resulting in enhanced N-myc stability.

### The positive-feedback loop of N-myc and INSM1 stimulate NB cell viability

**Figure 5:**
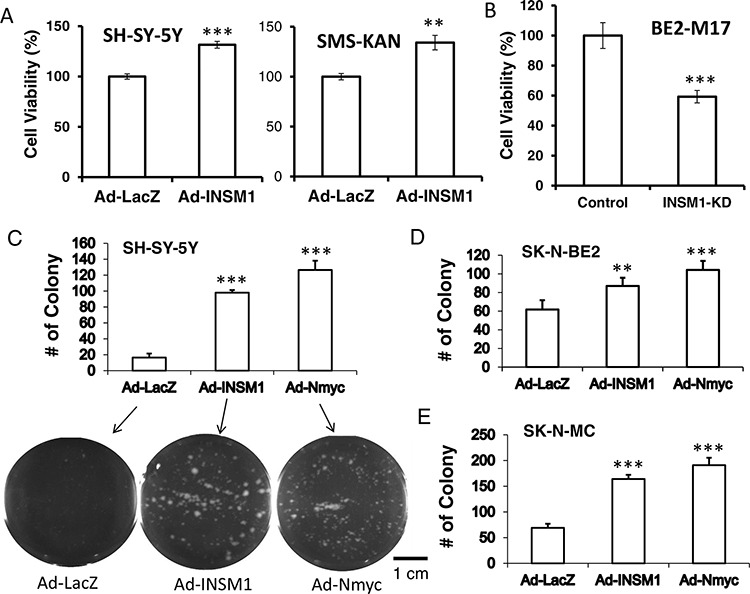
INSM1 stimulated NB cell growth and colony formation **A.** SH-SY-5Y and SMS-KAN cells were infected with Ad-LacZ or Ad-INSM1 virus for 48 h. **B.** BE2-M17 cells were transfected with INSM1 siRNA for 48 h. The infected or transfected cells were incubated in fresh culture medium for another 3 days. The cell viability was measured by a MTS assay. The cell viability means were calculated from 24 replicates. Data are represented as mean ± SEM. The statistically significant differences compared to the control group (CTRL) were noted as **P < 0.01, and ***P < 0.001. **C.** SH-SY-5Y, **D.** SK-N-BE2, and **E.** SK-N-MC cells were infected with Ad-LacZ, Ad-INSM1, or Ad-N-myc overnight. The next day, 5,000 infected cells were plated in soft agar for 3 weeks. The colonies formed were counted. Student's *t*-test of infected cells against Ad-LacZ control shows significant *p* value, ** < 0.01; *** < 0.001. SH-SY-5Y colony plates are shown underneath the graph.

We provide evidence that cross-talk between Shh, N-myc, and INSM1 play a crucial role in NB cell viability. *N-myc* is a well-known proto-oncogene and a downstream effector of Shh signaling during both normal and neoplastic cerebellar growth [21]. Over-expression of INSM1 increases cell viability in SH-SY-5Y and SMS-KAN cells whereas abrogating endogenous INSM1 expression in BE2-M17 cells decreases cell viability (Fig. 5A and 5B). We performed the INSM1-enhanced colony formation assay in SK-N-MC, SH-SY-5Y, and SK-N-BE2 cells. These three cell lines express either no INSM1/N-myc or low levels of INSM1/N-myc. Over-expression of INSM1 or N-myc dramatically increased colony formation in SH-SY-5Y, SK-N-MC, and SK-N-BE2 cells. Since the SK-N-BE2 cells express a small amount of endogenous INSM1 and N-myc, they displayed a less dramatic effect in the number of colonies formed. Our data reveals that INSM1 and N-myc facilitate cell viability and colony formation in NB cells (Fig. 5C, 5D, and 5E).

### INSM1 expression enhances cellular invasiveness and oncogenesis in NB cells

**Figure 6:**
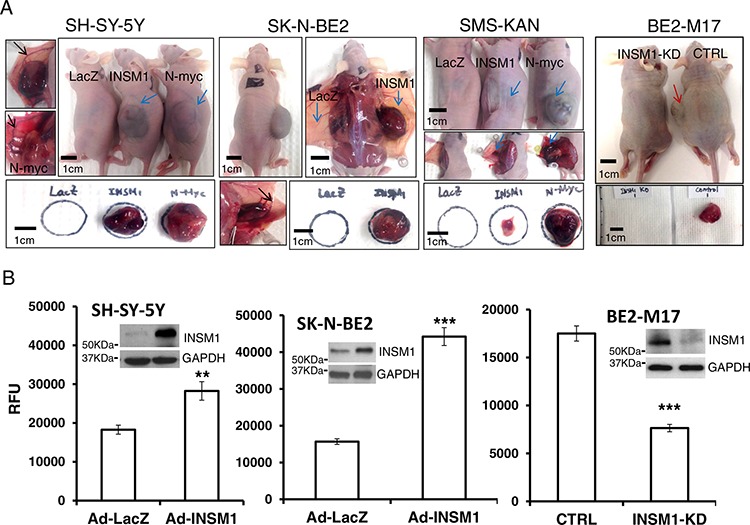
INSM1 promoted cellular invasion and enhanced tumorigenicity in NB tumors **A.** SH-SY-5Y, SK-N-BE2, SMS-KAN cells were infected with Ad-LacZ, Ad-INSM1 or Ad-N-myc virus for 48 h and BE2-M17 cells were transfected with INSM1 siRNA or control siRNA for 48 h. 1 × 10^7^ infected or transfected cells were subcutaneously injected in left side of *nu/nu* mice (*n* = 3) or Ad-LacZ infected cells were injected on the left side and Ad-INSM1 infected cells were inoculated on the right side of the *nu/nu* mouse. At least 3 mice were injected for each experimental group. The blue arrow indicates the Ad-INSM1, or Ad-N-Myc infected xenograft tumors. The red arrow indicates the control BE2-M17 xenograft tumor. The clear blood vessels surrounding the tumor mass in both INSM1- or N-myc induced tumors are shown by the black arrow. The individual tumors were harvested to shown underneath the photographs. **B.** SH-SY-5Y and SK-N-BE2 cells were infected with Ad-LacZ or Ad-INSM1 virus for 3 days, and BE2-M17 cells were transfected with INSM1 siRNA for 48 h. The infected or transfected cells were starved in culture medium without serum for another 16 h and then cell invasion was measured with a CultureCoat BME cell invasion assay. The invasive cells were labeled with Calcein AM and the fluorescence was quantified using a plate reader (RFU). The SEM was calculated from 8 replicates. The statistically significant differences were compared to the control group (Ad-LacZ or CTRL) and noted as **P < 0.01, and ***P < 0.001. The efficiency of over-expression or knockdown of INSM1 is shown above the graph in Western blot analysis.

The *in vitro* results showed that INSM1 promotes cell viability in NB cells. Further, an *in vivo* xenograft nude mouse tumor model revealed that INSM1 promoted NB tumorigenesis. We injected 1 × 10^7^ SH-SY-5Y, SMS-KAN, or SK-N-BE2 cells (*n* = 3) subcutaneously (s.c.), infected prior to injection with Ad-INSM1, Ad-N-myc, or Ad-LacZ virus for 48 hours. Alternatively, endogenous INSM1 in BE2-M17 cells was silenced by transfection with INSM1 siRNA or control scramble siRNA. After 2–3 months, except for one small pale tumor mass that was found in the mouse injected with Ad-LacZ infected SMS-KAN cells, no xenograft tumors were found in those mice s.c. injected with the other Ad-LacZ infected cells. Large bruised tumor masses were observed in the mice injected with INSM1 or N-myc over-expressing cell lines. The growth rate of the N-myc over-expression xenograft tumors was faster than the tumors over expressing INSM1 (Fig. 6A). Conversely, knock down of the endogenous *INSM1* gene expression in BE2-M17 cells inhibited tumor growth in the INSM1 KD group after 3 months while a tumor mass was found in the control group. These results support that INSM1 plays a critical role in enhancing tumorigenesis in NB. In addition, we noticed that while the xenograft tumor started forming, a flat bruised lesion was first found under the skin area where the Ad-INSM1 infected cells were injected. After the bruised spots were noticed, the solid tumor mass was observed for another 2–4 weeks. When we harvested the tumor tissues, obvious bloody clots and blood vessels (black arrow pointed) were observed around the tumors to support the nutrient supply to the tumor growth in those Ad-INSM1 and Ad-N-myc xenograft tumors (blue arrow pointed). These findings showed that INSM1 may promote angiogenesis like what is observed in N-myc induced NB tumors [25]. Thus, we hypothesize that INSM1 might play a role in cell migration and tumor angiogenesis. Cell invasion is a fundamental process required for tumor cell metastasis, angiogenesis, immune response, and embryonic development. Here, we use a BME-coated cell invasion assay to examine the effect of INSM1 on NB cell invasion. The enhanced xenograft tumor formation *in vivo* indicates that INSM1 is capable of promoting tumorigenesis. Over-expression of INSM1 increases cell invasion in SH-SY-5Y and SK-N-BE2 cells while abrogating endogenous INSM1 expression in BE2-M17 cells significantly decreased cell invasion (Fig. 6B). Both *in vitro* and *in vivo* data indicated that INSM1 is capable of stimulating NB cell proliferation, colony formation, invasion, and tumorigenesis. The mechanistic data strongly suggested that INSM1 is activated by N-myc and in turn INSM1 activates the PI3K/AKT signaling pathway to stabilize N-myc, which contributes to the increased malignancy of NB.

## DISCUSSION

In an *Insm1* global knockout mouse model, Insm1 was found to be a critical component in the transcriptional network responsible for the formation of SA lineage [15]. SA lineage cells are derived from neural crest which gives rise to sympathetic and adrenal chromaffin cells. The *Insm1* mutant mice showed a marked change in terminal differentiation of chromaffin cells and reduced the expression of genes whose protein products control catecholamine synthesis and secretion. *INSM1* gene is highly reactivated in NE tumors, which recapitulates the fetal NE tissue expression pattern. INSM1 expression was reported in childhood tumors including MB, retinoblastoma (RB), and NB [17, 18]. However, the functional effect of high INSM1 expression in NE tumors is not totally understood. Although the transcriptional activity of INSM1 is an important role for INSM1 during embryonic development, it is difficult to envision that de-regulation of INSM1 downstream target genes account for the NE transformation. Therefore, it is critical to identify how the INSM1 transcription factor contributes to NE cell transformation. Previous studies revealed that INSM1 possessed extra-nuclear activities that induced non-NE cell cycle arrest and enhanced the insulin receptor signaling pathway [22]. In this connection, we seek to uncover the molecular mechanisms underlying the functional role of INSM1 in NE tumor growth and transformation.

NB is the most common extracranial solid tumor in childhood and the most common cancer in infants. The etiology of NB is heterogeneous and not well understood. Blocking Shh signaling resulted in INSM1 down-regulation and reduced cellular viability in NB cells. Shh signaling is active in the development of the central nervous system [14]. The role of Shh signaling is widely studied in MB, NB, and other cancers [6–8]. Blocking Shh signaling led to cell growth retardation and inhibition of tumor development [26–28]. Here, we showed that Shh played a key role in up-regulating INSM1 expression in NB tumors. Using a Shh neutralizing antibody (5E1) or a Gli-dependent inhibitor (GANT-61) to block the Shh signaling pathway revealed that the INSM1/N-myc expression and the cell viability of NB decreased.

**Figure 7:**
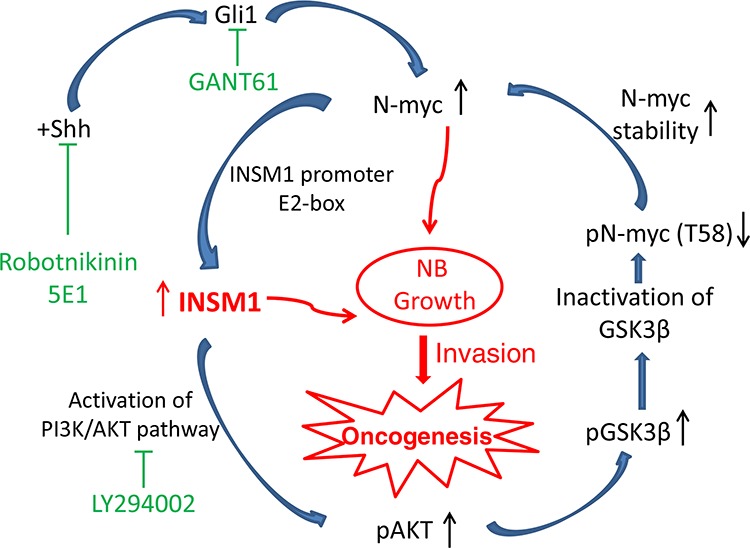
A model summarizing the INSM1 and N-myc positive-feedback loop that enhances NB cell growth, invasion, and oncogenesis N-myc amplified NB promotes aggressive tumor growth and correlates with a poor prognosis in patients [5]. Here, we identified that both the Shh signaling pathway and N-myc activate a novel downstream target gene, INSM1 that is crucial for the stability of N-myc protein accumulation through PI3K/AKT/GSK3β signaling pathways. This positive-feedback loop via the INSM1 expression enhances NB cell invasion and oncogenesis suggesting that the INSM1 NE factor is important in human NB transformation.

Nervous system malignant tumor cell proliferation and metastasis such as glioma, MB, and NB, are largely due to de-regulated N-myc expression [29–31]. The stabilization or degradation of Myc protein is controlled by the sequential phosphorylation of two key residues in the N-terminal Myc homology box I (MBI) region of *C-myc* (Thr-58 and Ser-62) and the equivalent sites in the mouse N-myc homolog of Thr-50 and Ser-54 [32]. We identified a novel positive-feedback loop mechanism via the INSM1 transcription factor that activated the PI3K/AKT signaling pathway, which subsequently induced phosphorylation of GSK3β kinase and blocked GSK3β kinase activity to stabilize N-myc. In the present study, we report that INSM1 has a critical function in stabilizing N-myc in NB tumor cells that express *N-myc* gene. Inversely, N-myc binds the E2-box region of the INSM1 promoter and activates *INSM1* gene expression. Although the functional role of INSM1 is a conventional nuclear transcription factor, it also possesses an extra-nuclear activity to activate the PI3K/AKT signaling pathway to block GSK3β activity and the N-myc (Thr-58) phosphorylation. Thr-58 phosphorylation leads to the stabilization of the N-myc protein and contributes to the transformation of NB tumors. To demonstrate the functional effects of INSM1 in NB, we showed that over-expression of INSM1 in NB cells induced cell growth and transformation. Conversely, knockdown of the endogenous INSM1 reduced the cell viability. Since INSM1 expression is predominantly in the poorly differentiated NB cells with a high potential for metastatic capacity, we investigated whether the presence of INSM1 contributes to NB invasion. The BME-coated invasion assay demonstrated that INSM1 directly correlated with the invasiveness of the NB tumor cells. Knockdown of INSM1 reduced invasive capacity of the N-myc amplified BE2-M17 cells. Furthermore, we examined the tumorigenicity potential of INSM1 in a xenograft nude mouse tumor model. INSM1 promoted the tumor growth. We observed that most of the INSM1-induced tumor growth was accompanied with blood vessel growth and a bruise indicating blood accumulation at the tumor location. Consistently, the N-myc over-expression nude mouse tumor model displayed a very similar pattern with massive vascular vessel growth. Thus, we demonstrated that INSM1 stimulates NB tumor cell growth through increasing the level of *N-myc* oncogene via increased stability. The molecular mechanisms revealed that N-myc and INSM1 formed a positive-feedback loop of regulation through the PI3K/AKT signaling pathways (Fig. 7). In this connection, the stabilized N-myc oncoprotein enhanced INSM1 expression and stimulated cell growth and tumorigenesis of NB.

## MATERIALS AND METHODS

### Cell culture and reagents

The human NB cell lines, SK-N-BE2, IMR-32, BE2-M17, SK-N-MC, SH-SY-5Y and SK-N-SH were obtained from the American Type Culture Collection. SMS-KAN was obtained from COG Cell Line and Xenograft Repository (CellLineInfo@cogcell.org). GANT-61 (10 mM) (MedChem Express) was prepared in DMSO as stock solution.

### MTS assay

3-(4,5-dimethyl-2-yl)-5-(3-carboxymethoxyphenyl)-2-(4-sulfophenyl)-2H-tetrazolium, inner salt (MTS) proliferation assay was carried out according to the manufacturer's protocol. In brief, each group of treated cells were collected and incubated in medium containing 20 ul of MTS reagent (Promega) at 37°C for 4 h. The assay was read absorbance at 490 nm using a 96-well plate spectrophotometer to calculate cell viability.

### Soft agar assay for colony formation

Soft agar was prepared with 1% bottom agar with 10% FBS. 5,000–10,000 cells were re-suspended in top 0.5% agar solution (2X RPMI and 5% FBS) and incubated at 37°C for 10 to 30 days.

### RNA isolation and analysis

RNA was extracted with TRIzol reagent and treated with 2 units of DNase I (Promega). cDNA synthesis using the High Capacity RNA-to-cDNA™ Kit (Life Tech.) was followed with the manufacturer's protocol. RNA was reverse-transcribed and analyzed by PCR and real-time PCR for the expression of INSM1, Smoothened, and N-myc. The relative RNA concentration of the target gene was normalized with GAPDH. Primers for INSM1: forward 5′-ACGGAATTCTGCCACCTGTGCCCAGTGTGCG GAGAG-3′, reverse 5′-CACCTCGAGCTAGCAGGCCGGGCGCACGG GCACCTGCAG-3′; Primers for Smoothened: forward 5′-GATGGGGACTCTGTGAGTGG-3′, reverse 5′-GGAAGCCAAAAATGCCCAGG-3′ and primers for N-myc: forward 5′-CCCTGAGCGATTCAGATGA-3′, reverse 5′-GACGCACAGTGATGGTGAAT-3′. The INSM1, N-myc primers and probes for real time PCR were purchased from Life Technologies.

### Promoter activity reporter assay

An 1.7 kb INSM1 promoter was inserted into a control promoterless pGL4.10 basic vector in front of luciferase 2 gene to construct the INSM1-promoter reporter plasmid. Transient transfection was performed with either the vehicle or INSM1 reporter into NB cells, SK-N-BE2, IMR-32, BE2-M17, SK-N-MC, and SK-N-SH with Fugene 6 (Bio-Rad) for 48 hours. The transfection efficiency was normalized with the TK-Renilla plasmid. Luciferase activities were measured using a Dual-Glo luciferase assay kit (Promega). Activities were averaged from three separate experiments.

### Chromatin immuno-precipitation (ChIP)

The ChiP sample was prepared by using the SimpleChIP® Plus Enzymatic Chromatin IP Kit (Cell Signaling Tech.) following the manufacturer's instruction. Anti-N-myc antibody (Santa Cruz Biotech.) was used in immuno-precipitation and histone-H3 antibody was used as a positive control. PCR was used to measure the relative abundance of the INSM1 gene promoter sequence enriched by a protein-specific immuno-precipitation versus a non-specific antibody control. The bound DNA was PCR amplified with primers spanning the human INSM1 promoter E2-box, forward 5′-GAGGAGAGACACAAAGCCCA-3′ and reverse 5′-TAGGTGCGGCAGATGTACCT-3′.

### Western blot analyses

Cell lysates were separated on 10% SDS-PAGE. The nitrocellulose membrane was blocked with 5% skim milk in TBST (20 mM Tris-HCl pH 7.6, 137 mM NaCl and 0.1% Tween-20), probed with the indicated primary antibody, such as anti-INSM1, anti-N-myc (Santa Cruz Biotech.), anti-pAKT, AKT (S-473), pGSK3β (S-9) and GSK3β (Cell Signaling Tech.), anti-phospho-Myc (pT-58) (Abgene) and anti-GAPDH at 4°C overnight and bound with HRP-conjugated secondary antibody (Bio-Rad) at room temperature for 1 h. A chemiluminescence substrate (Bio-Rad) was autographed onto a X-ray film.

### Cell invasion assay

INSM1 non-expressing (SH-SY-5Y, SMS-KAN) or low-expressing (SK-N-BE2) NB cells were infected with either Ad-LacZ or Ad-INSM1 virus, whereas INSM1 expressing BE2-M17 cells were subjected to the control scramble or INSM1 siRNA knockdown experiment. For the INSM1 knockdown study, BE2-M17 cells were transfected with INSM1 siRNA oligomers (5′-GGGAUCUGCUUAAAGUUUUtt-3′) using Lipofectamine RNAiMAX reagent (Life Technologies, San Francisco, CA). After 48 hours infection/transfection, the treated cells were cultured in serum-free medium for 16 h. Using Trevigen's CultureCoat cell invasion assay (Trevigen), the serum-starved infected or transfected cells (25,000 cell per well) were added to the top chamber and 150 μl of culture medium in bottom chamber. The culture was incubated in a 5% CO_2_ incubator at 37°C for 24 h. The cell invasion is quantified using Calcein AM/cell dissociation solution (100 μl) added to the bottom chamber of black receiver plate to label the invasive cells and then the fluorescence was detected with a 96-well fluorescence microplate reader (Synergy H1, Biotek).

### Xenograft tumor growth in nude mice

The transformation potential with INSM1 over-expression in NB cells was performed in a nude mouse tumor model with SH-SY-5Y, SK-N-BE2, and SMS-KAN cells (1 × 10^7^ cells). Cells were infected with Ad-INSM1, Ad-N-myc, or Ad-LacZ and were mixed 1:1 with Matrigel (high concentration, BD Biosciences) and injected subcutaneously (*n* = 3) into athymic NCR (*nu/nu*) mice (NCI). The BE2-M17 cells were transfected with INSM1 siRNA or scramble siRNA for 48 hours before injection. After 2–3 months, the NB tumors formed were harvested and the size of the xenograft tumors were measured. Animal procedures were performed in accordance with relevant guidelines and regulations. The IACUC committee from Louisiana State University Health Sciences Center approved the animal protocol.

### Statistical analysis

Values were corrected and expressed relative to an untreated control group. All experiments were repeated three times. Results are presented as mean ± SEM. Statistical analysis was performed using either the Student's *t*-test when only two groups were in the experiment or by an one-way ANOVA comparison of multiple groups using the Tukey-Kramer test with differences at *p* value of less than 0.05 being considered significant.

## Abbreviations

INSM1: insulinoma associated-1
BME: basement membrane extract
NB: neuroblastoma
MB: medulloblastoma
GB: glioblastoma
SMO: smoothened
RB: retinoblastoma
NE: neuroendocrine
SA: sympatho-adrenal
MTS: 3-(4,5-dimethyl-2-yl)-5-(3-carboxymethoxyphenyl)-2-(4-sulfophenyl)-2H-tetrazolium, inner salt
ChIP: chromatin immuno-precipitation
TBST: 20 mM Tris-HCl pH 7.6, 137 mM NaCl, and 0.1% Tween-20
